# Implementation of the A*rizona Pain and Addiction Curriculum*: Findings and Implications From a Statewide Evaluation

**DOI:** 10.3389/fpubh.2021.731016

**Published:** 2021-11-19

**Authors:** Lisa Villarroel, Aram S. Mardian, Evan Timme, Shakaib Rehman, Cara M. Christ

**Affiliations:** ^1^Division of Public Health Preparedness, Arizona Department of Health Services, Phoenix, AZ, United States; ^2^Chronic Pain Wellness Center, Phoenix VA Health Care System, Phoenix, AZ, United States; ^3^Department of Family, Community and Preventive Medicine, University of Arizona College of Medicine–Phoenix, Phoenix, AZ, United States; ^4^Tuberculosis Control Program, Arizona Department of Health Services, Phoenix, AZ, United States; ^5^Department of Education, Phoenix VA Health Care System, Phoenix, AZ, United States; ^6^Department of Internal Medicine, University of Arizona College of Medicine–Phoenix, Phoenix, AZ, United States; ^7^Department of Bioethics and Humanism, University of Arizona College of Medicine–Phoenix, Phoenix, AZ, United States; ^8^Arizona Department of Health Services, Phoenix, AZ, United States

**Keywords:** public health, pain, addiction, curriculum, evaluation, didactic dissonance

## Abstract

**Purpose:** The U.S. is struggling with dual crises of chronic pain and opioid overdoses. To improve statewide pain and addiction care, the Arizona Department of Health Services and 18 health education programs collaboratively created the evidence-based, comprehensive *Arizona Pain and Addiction Curriculum* which includes a Toolbox for Operationalization with adult learning theory applications and an annual program survey to assess curriculum implementation. The purpose of this study is to analyze the first year's survey data to better understand the implementation of a novel curriculum across all programs in the state.

**Materials and Methods:** Program surveys were sent 6 months after curriculum publication to all 18 health education programs in Arizona to assess the 6 Ds of curriculum implementation: Degree of implementation, Difficulty of implementation, Delivery methods, Faculty Development, Didactic dissonance and Discussion Opportunities.

**Results:** Responses from all program types (14/18 programs) indicated that there was widespread implementation of the curriculum, with 71% reporting that all ten Core Components had been included in the past academic year. The majority of programs did not find the Components difficult to implement and had implemented them through lectures. Seventy-seven percent of programs did not have a process to ensure clinical rotation supervisors are teaching content consistent with the curriculum, 77% reported not addressing student's didactic dissonance, and 77% of programs did not report asking students about their interactions with industry representatives.

**Conclusion:** In < 1 year after creation of the *Arizona Pain and Addiction Curriculum*, all program types reported wide implementation with little difficulty. This may represent a first step toward the transformation of pain and addiction education, and occurred statewide, across program types. Further focus on didactic dissonance, problem solving and faculty development is indicated, along with systematic education on pharmaceutical and industry influence on learners. Other programs may benefit from adopting this curriculum and may not experience significant challenges in doing so.

## Introduction

The United States is currently experiencing dual public health crises of chronic pain (1) and opioid-related overdoses (2). In light of the increasing opioid-related overdoses in Arizona, the governor of Arizona declared a statewide public health emergency on June 5, 2017 (3).

In response to this emergency, one of the recommendations from the Arizona Department of Health Services (ADHS) was to create a statewide, modern, evidence-based curriculum on pain and addiction for all prescriber types (4). Existing curricula included those created by Brown University (5) and the states of Massachusetts and Pennsylvania (6, 7), but these had too narrow a focus (e.g., on opioid use disorder only) or were for a single program type (e.g., medical schools). An Arizona Curriculum Workgroup, comprised of deans and curriculum representatives of all 18 medical, osteopathic, physician assistant, nurse practitioner, dental, podiatry and naturopathic programs in Arizona thus jointly created and published the *Arizona Pain and Addiction Curriculum* with its 10 Core Components (8).

The *Arizona Pain and Addiction Curriculum* vision is ambitious, as it seeks to redefine pain and addiction as complex, public health issues, requiring interprofessional care and involvement of systems. It focuses on intangible, upstream goals, such as reducing stigma, linking pain and addiction, demedicalizing chronic pain, increasing interdisciplinary care, and enhancing self and system's introspection. Tangible, downstream goals such as reducing opioid prescriptions or increasing the number of DATA-waivered providers able to prescribe buprenorphine for opioid use disorder are likely to follow from the cultural shift driven by the intangible goals (9).

The structure of the curriculum is designed to be comprehensive, adaptable, and practical for educators. All 10 Core Components are intended to be implemented as a whole, rather than a pick-and-choose approach. Each Component has cascading levels of detail (i.e., Component > Key Messages > Objectives > Detailed Content) for program types to be able to expand or contract the content detail (“accordion-style”) depending on the relevance. The curriculum also includes a Faculty Guide to aid with faculty development and a Toolbox for Implementation, both of which emphasize adult learning theory (10) as the pedagogical framework for the curriculum. Because of the transformational nature of the curricular content, the curricular materials encouraged the application of adult learning principles, including involving the learner in the evaluation of their instruction, having experience as the basis for learning activities, having material of immediate relevance to their future clinical practice, and incorporating the material into clinical case discussions. Each program could individualize the application of the material, but methodologies from the Toolbox (e.g., small group discussions, patient panels) were encouraged.

## Materials and Methods

### Development

ADHS and the curriculum workgroup worked together to develop program and learner evaluations of the *Arizona Pain and Addiction Curriculum* that would be administered annually.

The program evaluation collected information from each program on their progress, challenges and best practices of implementation. Although curriculum management and evaluation are considered in most accreditation standards, no such existing program evaluation was identified for a curriculum on pain and addiction, nor cross-program evaluations of pain and addiction curricula implementation. The curriculum developed in Massachusetts was reported to be implemented in all four medical schools, but no details on the assessment were provided.

The program evaluation was designed to assess curriculum implementation, with a goal of identifying successes and challenges in order to shape future curricular iterations. Given the public health interest in a statewide uptake in order to transform education and the provider practices that follow, questions were developed to assess the degree of implementation, difficulty of implementation and methods of content delivery. Given the novel material and approach, questions also included assessment of faculty development and exploration of learner's didactic dissonance (disconnect between what is taught and what is seen in practice). And last, given the role of the pharmaceutical industry on the opioid epidemic and the public health goal of preventing similar epidemics, questions were developed to assess programmatic approaches to learner's experience with industry.

Questions were refined by the curriculum workgroup during two in-person meetings in 2018 with consideration to relevance and brevity. The final program evaluation consisted of 20 questions (including multiple choice, Likert scales, and open-ended questions) that collected information about: Degree of implementation, Difficulty of implementation, Delivery methods (including those in the Toolbox for Operationalization), Faculty Development, Didactic dissonance and Discussion Opportunities (“6 Ds” of curriculum implementation) (see Appendix). Several questions touched on implementation of adult learning theories. The program evaluation was reviewed and edited by reviewers from Johns Hopkins and the Mayo Survey Research Center before administration (see Acknowledgments). The first program evaluation to be administered in 2019 was to serve as a baseline, presumably before the *Arizona Pain and Addiction Curriculum* was fully implemented across programs statewide.

The first learner evaluation was simultaneously administered in 2019 with a survey that collected information from learners about their knowledge, attitudes and plans for treating patients with pain and/or addiction, and these results will be presented in a separate paper.

### Administration

On April 4, 2019, the director of the Arizona Department of Health Services sent an email to the deans and curricular representatives of each health educational program in Arizona, requesting prompt and thorough completion of the program evaluation. On April 5, 2019 and May 15, 2019, links to the evaluation were sent out to the same program recipients by another physician at the Arizona Department of Health Services. The program evaluation was expected to be completed by one faculty member in each program familiar with the *Arizona Pain and Addiction Curriculum* by June 15, 2019.

Completion of the evaluation was not mandatory, but communication from the Arizona Department of Health Services stressed that strong, statewide data would benefit the state and its educational approach.

### Analysis

**Figure 1:**
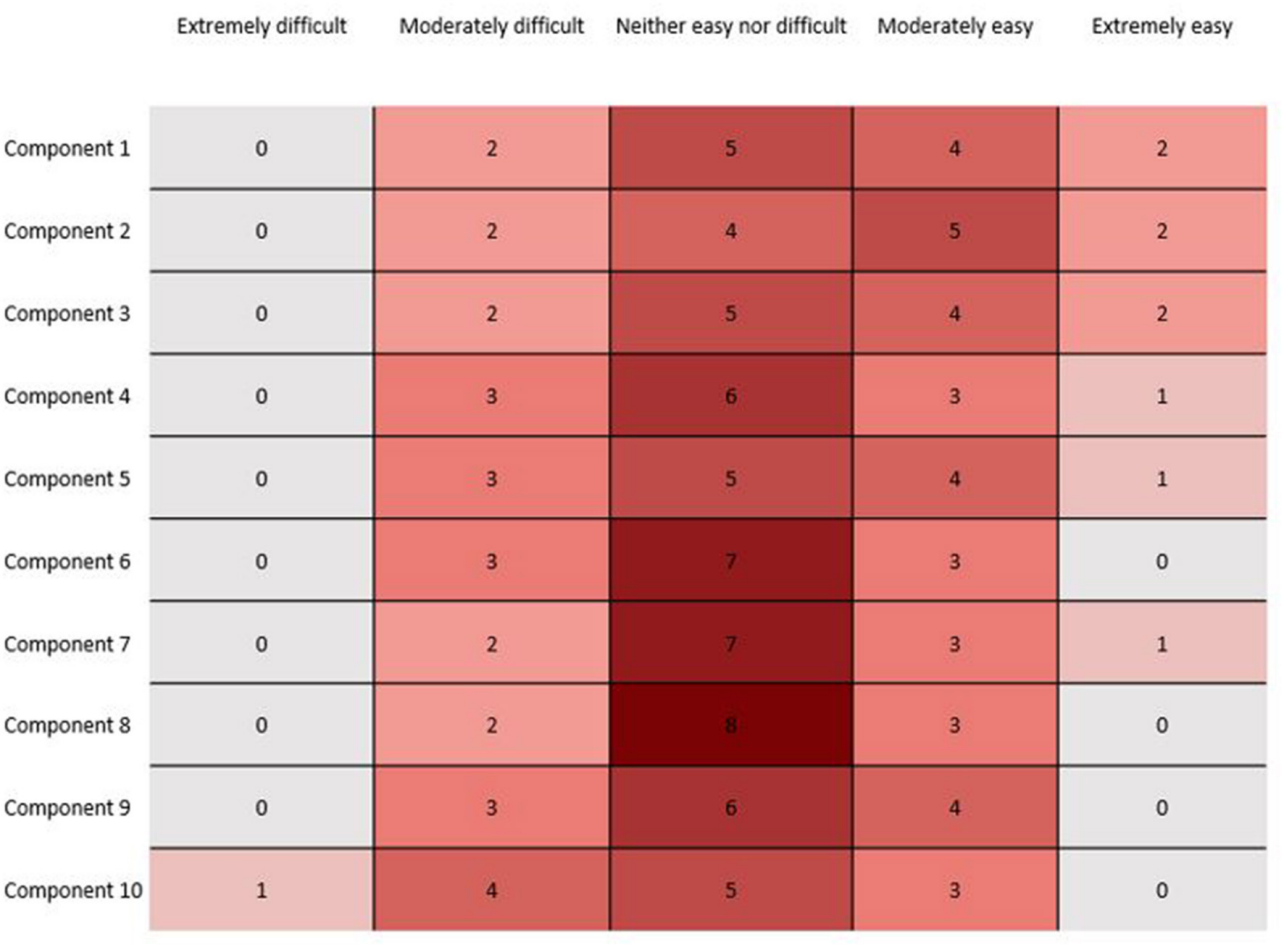
Heat graphic showing the difficulty of implementing each of the 10 Core Components of the *Arizona Pain* and *Addiction Curriculum*, as reported by programs.

Qualtrics software (Qualtrics, Provo, UT) was used to collect responses. Responses were processed and analyzed using SAS 9.4 (SAS Institute, Cary NC). Simple frequencies and percentages were completed for each response question. Additional processing was performed to produce interquartile and Heat Map findings in Figure 1.

## Results

### Statewide Implementation

In total, 14/18 Arizona health education programs completed the *Arizona Pain and Addiction Curriculum* program survey, representing 78% of programs in the state. Every type of health education program in Arizona (MD, DO, NP, PA, DMD, ND and DPM) was represented in the responses (Table 1).

**Table 1:** Name of Arizona program and degree that completed the *Arizona Pain and Addiction Curriculum* program evaluation.

**Name of Program**	**Degree**
Creighton University School of Medicine-Phoenix Regional Campus	MD
University of Arizona-College of Medicine Phoenix	MD
University of Arizona-College of Medicine Tucson	MD
A.T. Still University School of Osteopathic Medicine in Arizona	DO
Midwestern University-Arizona College of Osteopathic Medicine	DO
A.T. Still University School of Dentistry and Oral Health in Arizona	DMD
Northern Arizona University Physician Assistant Program	PA
A.T. Still University Physician Assistants Degree Program in Arizona	PA
Grand Canyon University College of Nursing and Health Care Professions	DNP
University of Arizona College of Nursing	DNP
Northern Arizona University Doctor of Nursing Practice	DNP
Arizona State University College of Nursing and Health Innovation	DNP
Southwest College of Naturopathic Medicine and Health Sciences	ND
Midwestern University-Arizona School of Podiatric Medicine	DPM

The number of students that received components of the *Arizona Pain and Addiction Curriculum* peaked in the second and third years of training (Table 2). Of note, not all programs contain a fourth year of training.

**Table 2:** Number of students from all program types receiving components of the *Arizona Pain and Addiction Curriculum*, as reported by 14 programs.

**Program year**	**Number of students**
1	838
2	1,013
3	978
4	706

### Degree of Implementation

10/14 (71%) of programs responded that all ten Core Components of the *Arizona Pain and Addiction Curriculum* were included in their curriculum in the past academic year. Within the options of Fully Implemented, Partially Implemented or Not at All Implemented, 50% or more of programs reported having fully implemented all but one of the ten Components (Table 3).

**Table 3:** The 10 Core Components of the *Arizona Pain* and *Addiction Curriculum* and (%) of schools reporting full implementation of each.

	**Component Description**	**Number of schools that reported fully implemented of the component (%)**
1	Define pain and addiction as multidimensional, public health problems.	10/14 (71%)
2	Describe the environmental, healthcare systems and care model factors that have shaped the current opioid epidemic.	8/14 (57%)
3	Describe the interrelated nature of pain and opioid use disorder, including their neurobiology and the need for coordinated management.	9/14 (64%)
4	Use a socio-psycho-biological model to evaluate persons with pain and opioid use disorder.	7/14 (50%)
5	Use a socio-psycho-biological model to develop a whole-person care plan and prevention strategies for persons with pain and/or opioid use disorder.	9/14 (64%)
6	Reverse the unintended consequences created by the medicalization of chronic pain by empowering persons with self-management strategies for persons with pain and/or opioid use disorder.	7/14 (50%)
7	Use and model language that destigmatizes, reflects a whole-person perspective, builds a therapeutic alliance and promotes behavior change.	6/14 (43%)
8	Employ an integrated, team-based approach to pain and/or addiction care.	7/14 (50%)
9	Engage family and social support in the care of pain and/or addiction.	7/14 (50%)
10	Critically evaluate systems and seek evidence-based solutions that deliver quality care and reduce industry influence in the treatment of pain and opioid use disorder.	7/14 (50%)

### Difficulty of Implementation

Thirteen of the 14 programs completed the implementation section. The majority of programs reported that Components were not difficult to implement. Of the 130 total component responses, 103/130 (79%) were reported as “Extremely easy,” “Moderately easy,” or “Neither easy nor difficult. Component 10 had the greatest level of reported difficulty by programs; more than one-third of responding programs indicated that Component 10 was “Extremely difficult” or “Moderately difficult” to implement (Figure 1). Although the free-text answers did not specifically reference the difficulty of Component 10, they did mention struggles with faculty development, culture change, and finding sufficient time required to teach the material. On the other hand, there were also comments that implementation was not difficult and that the curriculum and accompanying Faculty Guide were clear.

### Delivery Methods

All responding programs reported implementing Components through lectures (7/13, 54%), online modules (2/13, 15%), or a combination of both lecture and online modules (4/13, 31%). In the free-text section asking for best practices, programs reported successful models of partnering with addiction clinics and specifically choosing to focus on pain and addiction content during the clerkship years.

### Faculty Development

**Figure 2:**
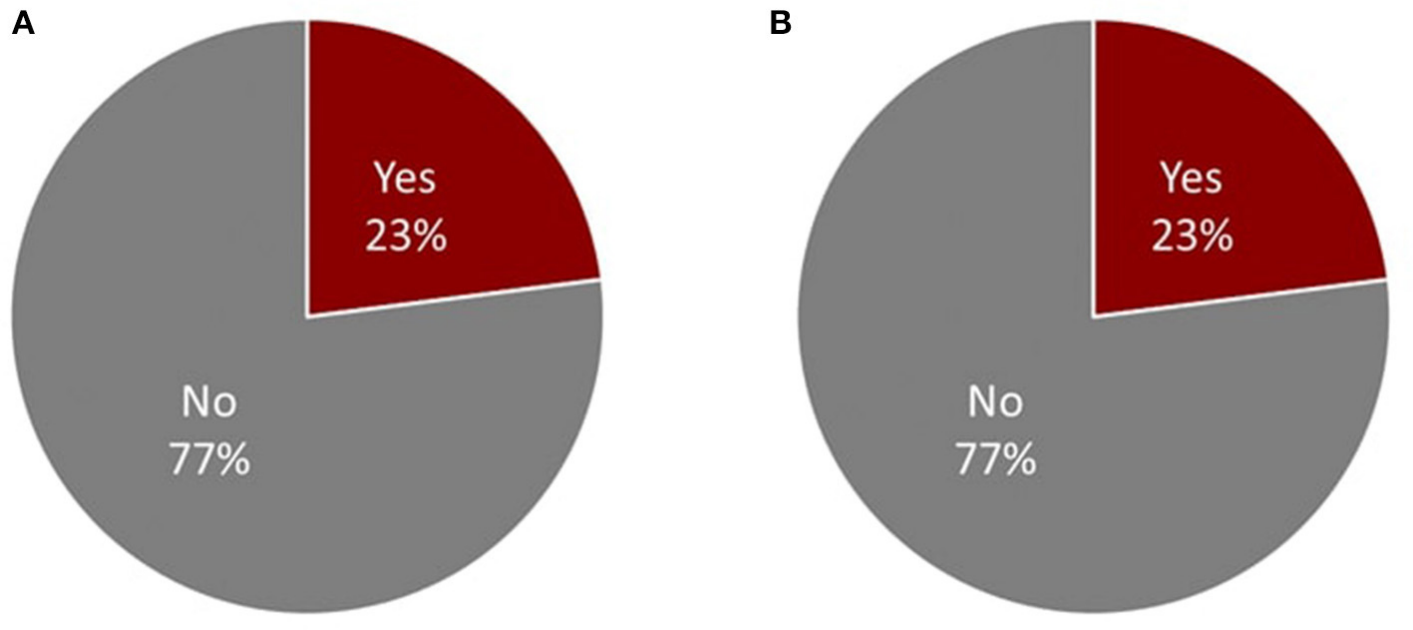
Percentage of respondents (*n* = 14) responding yes or no to **(A)** “Do you have a process for ensuring clinical rotation supervisors are consistent and able to implement the *Arizona Pain and Addiction Curriculum*?” and **(B)** “Following clinical rotations are, students asked about their observations of pain and addiction care, and how it may or may not differ from the *Arizona Pain and Addiction Curriculum*?”

77% (10/13) of programs reported not having a process to ensure clinical rotation supervisors are teaching content consistent with the *Arizona Pain and Addiction Curriculum* (Figure 2).

### Didactic Dissonance

77% (10/13) of programs reported not discussing students' observations after clinical rotations about pain and addiction care, and how it may or may not differ from what was taught in the *Arizona Pain and Addiction Curriculum* (Figure 2).

### Discussion Opportunities

Programs reported 2,407 total students having received specific didactic training about the potential influence of industry on practice, with the plurality in Year 2 (Year 1-566; Year 2-873; Year 3-598; Year 4-370).

However, most programs did not report specifically asking students about their interactions with industry representatives upon completion of clinical rotations (77%, 10/13). In the free-text section asking about unique examples of training on industry influence, several programs wrote that they did not have any; another suggested that students are welcome to interact with industry with their preceptor's approval and the program “[does] not seek to control that.” One osteopathic program described having a specific lecture about industry influence during orientation and embedding further industry topics during Pharmacology units.

## Discussion

*The Arizona Pain and Addiction Curriculum* is a novel initiative and its joint creation with all types of health educational program in a state is unprecedented. However, achieving a transformation of care for patients with pain and/or addiction requires implementation of the curriculum and integration of the material into the lectures, as well as patient-level, community-level, and clinical forms of education. This appears to have been done with varying success, limited primarily by faculty development and time.

### Statewide Implementation

Program participation in the baseline evaluation was notable and included the majority of programs in the state and representation from every program type. Over 2,000 students received at least some component of the curriculum already by spring 2019, suggesting widespread implementation across Arizona.

### 6 Ds (Degree, Difficulty, Delivery Method, Faculty Development, Didactic Dissonance, Discussion Opportunities)

It was unexpected to find that most programs reported having fully implemented each Component of the Curriculum. This either reflects program's unexpectedly strong momentum to make curriculum changes or that the questions were phrased broadly as to ask about the primary concept of each Component rather than the more nuanced Objectives under each Component. Results from future evaluations may help to clarify this and what “fully implemented” means to each program.

Most programs did not find the *Arizona Pain and Addiction Curriculum* difficult to implement. The highest difficulty ratings for Component 10 implementation is understandable yet unfortunate, as skepticism and solving problems at a systems-level are difficult to canonize but essential for future improvement.

One identified area of growth is the need to implement adult learning principles. The use of lectures for all components was predominant, with fewer programs reporting use of standardized patients, small group activities, or other examples from the Toolbox of Operationalization. This is important given the transformative nature of the curriculum and the recognition that more active learning approaches result in more profound levels of internalization and integration for learners. Recognizably, these approaches such as small group discussions, interactions with patient panels (patients recovering from pain and/or addition), and rotations with community mutual support groups (e.g., Alcoholics Anonymous or Narcotics Anonymous) are more difficult for programs to organize and maintain than a single didactic lecture.

Most schools did not have a system in place to ensure that faculty were familiar and teaching concepts in line with the *Arizona Pain and Addiction Curriculum*, but we hope this improves over time. Faculty development and familiarity with the new and transformative concepts in the curriculum is key to its success, and the Faculty Guide was created to help with that. Annual meetings with the curriculum workgroup will guide the production of further tools to assist faculty comfort with the new material. It remains to be seen the degree of engagement the faculty will have with the comprehensive material presented in the Faculty Guide and methods recommended in the Toolbox for Operationalization.

There is also an opportunity for programs to delve deeper into the concept of “didactic dissonance.” This curriculum reflects a modern approach to pain and addiction and a cultural transformation from prior teaching, and learners are expected to experience some degree of disconnect between what is taught in the curriculum and what they see in practice. By including the concept of didactic dissonance in the Toolbox for Operationalization, the curriculum aims to turn this ostensible barrier to education into an opportunity to deepen and reinforce learning and cultural transformation. Others have published on the hidden curriculum's exposure to learners (11) and the American College of Physicians recommends that faculty “encourage reflection and discussion of positive and negative behaviors in the training environment” (12). The impact of this curriculum is likely to be diminished if the didactic dissonance is not openly addressed.

Similarly, addressing the impact of the pharmaceutical and device industries on students during their experience on clinical rotations, particularly with external preceptors where school policies on pharmaceutical influence are not observed, would be beneficial. Contact with industry influences trainee and health professional behavior (13) and was an important factor in the development of the opioid epidemic (14). Without circling back to discuss experiences that students have with industry, this is likely a missed opportunity to reset standards. There were very few examples of best practices on pharmaceutical relationship education; only one osteopathic program actively addressed industry influence throughout their 4 year curriculum.

### Methodological Limitations

One limitation on this study includes the timing of the evaluation. Different health education programs in Arizona vary in terms of starting and ending dates and may have affected the degree of program implementation.

Another limitation is response bias, as the respondents were not blinded. It is possible that the programs wanted to show how their program was “putting its best foot forward.”

A final limitation is the lack of standardization of who completed the evaluation. Anecdotal reports of who completed the evaluation on behalf of the program ranged from the original curriculum workgroup to other deans that had not participated in the development or implementation. The evaluation was straightforward and rapid if the person completing it was familiar with the Curriculum; it was onerous and long if the person was not. This may have impacted the quality of results.

## Future Directions and Conclusion

Overall, there was remarkable uptake of the *Arizona Pain and Addiction Curriculum* by educational programs in Arizona in a short time period. Understanding that this is a comprehensive curriculum, it is unlikely to be successful as an “add-on” but requires a longitudinal integration of the material through the training years. It is possible that some of the older teaching material on pain (e.g., with a primary focus on biological factors) and addiction (e.g., with a primary focus on disciplinary efforts for providers) could be replaced with the modern, evidence-based, transformational material of the *Arizona Pain and Addiction Curriculum*. The deans and the curriculum representatives from the Curriculum Workgroup continue to meet annually to discuss implementation; we expect this to be an iterative process of finding and refining best practices.

*The Arizona Pain and Addiction Curriculum* was jointly created by all 18 health educational programs in Arizona, and there is evidence of widespread uptake in all program types, with little difficulty. Further focus on the adult learning principles around didactic dissonance and problem solving is likely indicated, along with faculty development and specific educational programs addressing the influence of pharmaceutical and device industries on provision of care. This evaluation will continue to be administered on a yearly basis, and we look forward to seeing how implementation and feedback changes over time. Progress on program implementation is a first step toward transforming education, and its impact on learners will be measured and presented in future manuscripts. The curriculum and its evaluation are free to access and non-proprietary; other schools may benefit from adopting this curriculum and its supportive features of a Faculty Guide and Toolbox for Operationalization.

## Data Availability Statement

The datasets presented in this article are not readily available because there is not currently a publicly available dataset. Requests to access the datasets should be directed to pio@azdhs.gov.

## Ethics Statement

Ethical review and approval was not required for the study on human participants in accordance with the local legislation and institutional requirements. Written informed consent for participation was not required for this study in accordance with the national legislation and the institutional requirements.

## Author Contributions

LV and AM were involved in conceptualization, methodology, project administration, supervision, and manuscript writing and editing. ET was involved in data curation, formal analysis, and manuscript review and editing. SR was involved in manuscript review and editing. CC was involved in conceptualization, project oversight, supervision, and manuscript review and editing. All authors contributed to the article and approved the submitted version.

## Funding

The funding for the layout and publication of the *Arizona Pain and Addiction Curriculum* comes from Arizona's State Opioid Response Grant. The funding for the publication fees will come from the Arizona State Opioid Response Grant.

## Conflict of Interest

The authors declare that the research was conducted in the absence of any commercial or financial relationships that could be construed as a potential conflict of interest.

## Publisher's Note

All claims expressed in this article are solely those of the authors and do not necessarily represent those of their affiliated organizations, or those of the publisher, the editors and the reviewers. Any product that may be evaluated in this article, or claim that may be made by its manufacturer, is not guaranteed or endorsed by the publisher.
