# Supplementary material for: Implementation of the Arizona Pain and Addiction Curriculum: Findings and Implications From a Statewide Evaluation
Source: Front Public Health. 2021 Nov 19;9:731016. doi: 10.3389/fpubh.2021.731016 (PMC8641291; doi:10.3389/fpubh.2021.731016)
Supplement: Supplementary file 1 [file Table_1.DOCX]

**APPENDIX**

**The Arizona Pain and Addiction Curriculum’s Program Survey**

**Introduction**

To the Program Representative:

Thank you for your participation in the statewide annual evaluation of the *Arizona Pain and Addiction Curriculum* implementation.

- This survey should take about 10-15 minutes to complete.
- This survey can be completed on a computer or a phone.
- It will be helpful to have your Curriculum close by, so you can quickly refer to Components 1-10. For reference, these are also available at [www.azhealth.gov/curriculum](about:blank).
- All answers will be collected securely and maintained at the Arizona Department of Health Services.
- Findings will be shared with the curriculum workgroup meeting June 25, 2019.
- Participation in this survey and workgroup meeting is voluntary.
- This survey should be completed by June 1, 2019.

**Program Demographics**

**Q1**

o Your Name: ________________________________________________

o Your Position: ________________________________________________

**Q2** Name of Educational Institution: [Select name of institution]

**Q3** Program Type:

o MD

o DO

o NP

o PA

o DMD

o DPM

o ND

**Q4** How many 1st, 2nd, 3rd (± 4th) year students received components of the *Arizona Pain and Addiction Curriculum* this past academic year?

o 1st year ________________________________________________

o 2nd year ________________________________________________

o 3rd year ________________________________________________

o 4th year ________________________________________________

**Q5** How many students do you anticipate graduating from your program this academic year?___________________________________________________________

**Implementation of Core Components**

**Q6** Were all ten Core Components of the *Arizona Pain and Addiction Curriculum* included in your program’s curriculum this past academic year?

o Yes

o No

**Q7** How fully were Core Components (1-10) implemented as part of your program’s

curriculum this past academic year?

|  | Not Implemented | Partially Implemented | Fully Implemented |
| --- | --- | --- | --- |
| **Component 1:** Define pain and addiction as multidimensional, public health problems. | o | o | o |
| **Component 2:** Describe the environmental, healthcare systems and care model factors that have shaped the current opioid epidemic and approach to pain care. | o | o | o |
| **Component 3:** Describe the interrelated nature of pain and opioid use disorder, including their neurobiology and the need for coordinated management. | o | o | o |
| **Component 4:** Use a socio-psycho-biological model to evaluate persons with pain and opioid use disorder. | o | o | o |
| **Component 5:** Use a socio-psycho-biological model to develop a whole-person care plan and prevention strategies for persons with pain and/or opioid use disorder. | o | o | o |
| **Component 6:** Reverse the unintended consequences created by the medicalization of chronic pain by empowering persons with self-management strategies, and include an awareness of chemical coping. | o | o | o |
| **Component 7:** Use and model language that destigmatizes, reflects a whole-person perspective, builds a therapeutic alliance and promotes behavior change. | o | o | o |
| **Component 8:** Employ an integrated, team-based approach to pain and/or addiction care. | o | o | o |
| **Component 9:** Engage family and social support in the care of pain and/or addiction. | o | o | o |
| **Component 10:** Critically evaluate systems and seek evidence-based solutions that deliver quality care and reduce industry influence in the treatment of pain and opioid use disorder. | o | o | o |

**Q8** How were the following Core Components implemented into the program and in what year(s) of training was each one taught? Choose multiple, if applicable.

|  |  | | | | |  | | | |
| --- | --- | --- | --- | --- | --- | --- | --- | --- | --- |
|  | Lectures | Online Module | Standardized Patients | Small Group Activity | Other | *Year 1* | *Year 2* | *Year 3* | *Year 4* |
| **Component 1** | ▢ | ▢ | ▢ | ▢ | ▢ | ▢ | ▢ | ▢ | ▢ |
| **Component 2** | ▢ | ▢ | ▢ | ▢ | ▢ | ▢ | ▢ | ▢ | ▢ |
| **Component 3** | ▢ | ▢ | ▢ | ▢ | ▢ | ▢ | ▢ | ▢ | ▢ |
| **Component 4** | ▢ | ▢ | ▢ | ▢ | ▢ | ▢ | ▢ | ▢ | ▢ |
| **Component 5** | ▢ | ▢ | ▢ | ▢ | ▢ | ▢ | ▢ | ▢ | ▢ |
| **Component 6** | ▢ | ▢ | ▢ | ▢ | ▢ | ▢ | ▢ | ▢ | ▢ |
| **Component 7** | ▢ | ▢ | ▢ | ▢ | ▢ | ▢ | ▢ | ▢ | ▢ |
| **Component 8** | ▢ | ▢ | ▢ | ▢ | ▢ | ▢ | ▢ | ▢ | ▢ |
| **Component 9** | ▢ | ▢ | ▢ | ▢ | ▢ | ▢ | ▢ | ▢ | ▢ |
| **Component 10** | ▢ | ▢ | ▢ | ▢ | ▢ | ▢ | ▢ | ▢ | ▢ |

**Q8a** If other, please specify. __________________________________________________

**Q9** How difficult was it to implement each Core Component?

|  | Extremely difficult | Moderately difficult | Neither easy nor difficult | Moderately easy | Extremely easy |
| --- | --- | --- | --- | --- | --- |
| Component 1 | o | o | o | o | o |
| Component 2 | o | o | o | o | o |
| Component 3 | o | o | o | o | o |
| Component 4 | o | o | o | o | o |
| Component 5 | o | o | o | o | o |
| Component 6 | o | o | o | o | o |
| Component 7 | o | o | o | o | o |
| Component 8 | o | o | o | o | o |
| Component 9 | o | o | o | o | o |
| Component 10 | o | o | o | o | o |

**Q10** Please describe why a specific (or multiple) component(s) were challenging to teach or implement. _______________________________________________________________

**Q11** What unique example(s) or successful models of training would you like to share regarding implementation of the *Curriculum*? ___________________________________

**Faculty Development**

**Q12** How many representatives from your program attended the Arizona Pain and Addiction Curriculum Summit (November 2018)? _____________________________________

**Q13** Of the representatives who attended the Arizona Pain and Addiction Curriculum Summit (November 2018), what are their roles in your program? Choose all that apply.

o Curriculum Leadership

o Teaching Faculty

o Community Faculty or Staff

o Administrative Support

o Other ________________________________________________

**Q14** Do you have a process for ensuring clinical rotation supervisors are consistent and able to implement the *Arizona Pain and Addiction Curriculum*?

o Yes

o No

**IF = YES;**

**Q14a** Please explain. __________________________________________________

**Q15** Following clinical rotations, are students asked about their observations of pain and addiction care, and how it may or may not differ from the *Arizona Pain and Addiction Curriculum*?

o Yes

o No

**IF = YES;**

**Q15a** Please explain._____________________________________________________

**Industry Influence**

**Q16** How many students last year received specific didactic training about the potential influence of industry (e.g. pharmaceutical companies, international/device companies, supplement companies) on clinical practice?

o Year 1 ________________________________________________

o Year 2 ________________________________________________

o Year 3 ________________________________________________

o Year 4 ________________________________________________

**Q17** Upon completion of hospital and community rotations, are students specifically asked about their interaction with industry representatives?

o Yes

o No

**IF= Yes**

**Q18** Please explain.__________________________________________________

**Q19** What unique example(s) or successful models of training do you have regarding implementation of the *Arizona Pain and Addiction Curriculum* on the topic of industry influence? ____________________________________________________________

**Feedback**

**Q20** Please provide additional comments on your experience teaching components of the *Arizona Pain and Addiction Curriculum* this past year, including potential impact on learners, faculty and patient care. ________________________________________________

**Close**

Thank you for participating in this survey. We look forward to seeing you June 25, 2019 for our annual curriculum workgroup meeting!
